# The Importance of the “Damage Control” Strategy in Multiple Organ Injuries, Pathophysiology and Principles of Hemorrhage Control

**DOI:** 10.3390/jcm15072549

**Published:** 2026-03-26

**Authors:** Oliwia Klimek, Jakub Dudek, Anna Czesyk, Bartosz Sierant, Wiktoria Górecka, Grzegorz Gogolewski, Tomasz Jurek, Zuzanna Ochocka, Amelia Jankowska

**Affiliations:** 1 Faculty of Medicine, Wroclaw Medical University, 51-601 Wroclaw, Poland; oliwia.klimek11@icloud.com (O.K.); aniaczesyk28@wp.pl (A.C.); bartoszsierant@wp.pl (B.S.); zuzanna.ochocka@student.umw.edu.pl (Z.O.); amelia.jankowska@student.umw.edu.pl (A.J.); 2 Student Scientific Association of Emergency and Critical Care Medicine, Wroclaw Medical University, Borowska 213, 50-556 Wroclaw, Poland; 3 Lower Silesian Specialist Hospital—Centre for Emergency Medicine, ul. Gen. Augusta Emila Fieldorfa 2, 54-049 Wroclaw, Poland; 4 University Centre of Dentistry, Krakowska 26, 50-425 Wroclaw, Poland; wiktoriagorecka@gmail.com; 5 Clinical Department of Emergency Medicine, Faculty of Medicine, Wroclaw Medical University, Borowska 213, 50-556 Wroclaw, Poland; grzegorz.gogolewski@umw.edu.pl; 6 Battlefield Medicine and Forensic Ballistics Laboratory, Department of Forensic Medicine, Faculty of Medicine, Wroclaw Medical University, Jana Mikulicza-Radeckiego 4, 50-345 Wroclaw, Poland; tomasz.jurek@umw.edu.pl; 7 Emergency Department, Jan Mikulicz Radecki University Hospital in Wroclaw, Borowska 213, 50-556 Wroclaw, Poland

**Keywords:** Damage Control Resuscitation, multiple organ injuries, hypothermia, coagulopathy, hemorrhage control, blood products, trauma care, resuscitation strategies, shock management, transfusion therapy

## Abstract

**Background/Objectives**: Damage Control Resuscitation (DCR) is a critical strategy in the management of severe trauma, focusing on the optimisation of the patient’s physiological condition. This study reviews current DCR strategies, emphasizing the mitigation of the “diamond of death”—hypothermia, acidosis, coagulopathy, and hypocalcemia—while addressing complex disturbances like respiratory distress syndrome (ARDS) and (acute kidney injury) AKI in high-ISS (Injury Severity Score) patients. **Methods**: A systematic review of 59 contemporary sources was conducted, encompassing clinical trials (e.g., CRASH-2), military-to-civilian protocol translations, and guidelines from the C and European Resuscitation Council. The analysis focused on pre-hospital interventions, in-hospital transfusion protocols, and the impact of transport logistics on survival. **Results**: Evidence highlights that aggressive crystalloid resuscitation (over 5 L) significantly increases mortality, favoring balanced blood component therapy (1:1:1 ratio) or Whole Blood guided by viscoelastic testing like rotational thromboelastometry (ROTEM) or thromboelastography (TEG). Pre-hospital success is driven by rapid hemorrhage control via tourniquets, early administration of Tranexamic Acid (TXA), no aggressive crystalloids, permissive hypotension, proactive calcium supplementation is recommended in early care. Furthermore, the integration of Helicopter Emergency Medical Services (HEMS) is independently associated with improved survival in multi-organ trauma by reducing time to definitive care and facilitating “en-route” damage control. **Conclusions**: The evolution of rescue strategies focused on mitigating the effects of the diamond of death, combined with the implementation of permissive hypotension and optimized HEMS logistics, constitutes the foundation of a modern model aimed at minimizing mortality in multi-organ trauma.

## 1. Introduction

DCR is a systematic approach to major trauma that integrates the <C>ABC paradigm—catastrophic bleeding, airway, breathing, and circulation—with a series of clinical interventions, from the point of injury to definitive care. The primary objective of DCR is to minimise blood loss, maximise tissue oxygenation, and enhance patient outcomes. In recent years, DCR has gained increasing relevance as a cornerstone of trauma care. Haemorrhage continues to be the leading cause of preventable death, accounting for 30% to 40% of all trauma-related fatalities [1]. As a therapeutic strategy, DCR specifically targets the pathophysiological conditions that exacerbate haemorrhage in trauma patients [2]. The concept of DCR was first introduced within the UK Defence Medical Services in 2007, amalgamating advancements in both pre-hospital and hospital-based trauma care into a unified clinical doctrine. Since its inception, DCR has also been adopted as a framework for tactical medical planning in contemporary military operations, enhancing its global applicability and relevance [1].

## 2. Pathophysiology of Multiple Trauma

Multiorgan injuries (polytrauma) refer to trauma involving two or more organ systems, which results in profound physiological disturbances and carries a significant risk of mortality. The pathophysiology underlying these injuries is multifaceted, encompassing a range of intricate mechanisms that disrupt normal bodily functions, triggering a cascade of systemic effects that may compromise overall health and organ function [3,4].

### 2.1. Classification of Multiple Trauma—Mechanisms of Injury

Multiorgan injuries can be classified into several categories. These include classifications based on the mechanism of injury—blunt or penetrating trauma—and the dynamics of the injury, which distinguishes between high-energy injuries (e.g., traffic accidents) and low-energy injuries (e.g., falls from standing height). Additionally, they can be categorised according to the specific organ systems involved [3,4].

In clinical practice, the severity of multiorgan injuries is often assessed using the ISS and the Abbreviated Injury Scale (AIS) [3,4].

Traffic accidents and falls from height are the most common causes of such injuries, with the most frequently affected components being bone fractures, head injuries, and thoracic injuries. A study by Dziubiński et al., comparing data from 2007 and 2015, revealed a shift in the frequency of injuries by body region in multiorgan trauma. Specifically, head injuries increased by 14%, chest injuries decreased by 21%, and bone fractures decreased by 11%. This shift coincided with a 10% improvement in patient survival over the 8-year period [3].

Multiorgan injuries commonly result from blunt or penetrating trauma. A study by Donohue et al. compared mortality rates between patients with blunt and penetrating trauma, evaluating 24 h mortality, 30-day mortality, and length of stay in the intensive care unit. In all of these parameters, patients with blunt trauma had demonstrated significantly worse outcomes [4].

From a pathophysiological standpoint, thoracic and soft tissue injuries are particularly critical components of multiorgan trauma [5,6,7].

Thoracic trauma encompasses a range of injuries, with pulmonary contusion being the most common, followed by hemothorax or even lung laceration. These injuries lead to damage to lung tissue and blood vessels, resulting in bleeding into the bronchoalveolar space and lung parenchyma. This bleeding impairs gas exchange, and the accumulation of blood in the lungs and pleural cavity activates the coagulation cascade, promoting clot formation. Consequently, pulmonary perfusion is further compromised, exacerbating the disruption of gas exchange. Moreover, in cases of severe thoracic trauma, attention must be given to the scenario where at least three consecutive ribs are fractured in two or more places, resulting in flail chest. This condition impairs respiratory mechanics, causing ventilation-perfusion mismatch, which can ultimately progress to ARDS [5,6].

In a study by Kapicibasi analyzing 130 cases of blunt thoracic trauma, it was found that falls (61.3%) were the leading cause of injury in elderly individuals (>65 years), while traffic accidents were the predominant cause in younger individuals (50%). Additionally, the occurrence of flail chest was significantly higher in the elderly cohort, and their average hospital stay was considerably longer. These findings underscore the necessity of addressing multiorgan trauma with particular focus on elderly patients [5,6].

Soft tissue trauma induces local hypoxia, which impairs aerobic respiration and leads to tissue hypoxia and necrosis. In response, platelets are activated, releasing mediators that stimulate macrophages and fibroblasts to produce granulation tissue. Over time, vascular tissue renewal occurs at the site of injury through the process of angiogenesis [5,6,7].

Crush injuries involving soft tissues are particularly noteworthy. These injuries result from compressive forces that cause direct damage to soft tissues, muscles, bones, nerves, blood vessels, and other structures, depending on the injury site. The upper limbs are the most commonly affected, followed by the lower limbs and torso. A key phenomenon in the progression of these injuries is the so-called “second-hit phenomenon.” In addition to the localized inflammatory response, crush injuries frequently lead to rhabdomyolysis, which, depending on the extent of muscle damage, can progress to AKI. Renal failure is a frequent complication, with a mortality rate of up to 20% [5,6,7].

### 2.2. Cellular-Level Mechanisms

When multiple trauma occurs, pathophysiological changes begin at the cellular level, ultimately responsible for a self-perpetuating cycle that can culminate in multi-organ failure. Regardless of the injury mechanism, but depending on its severity (extent of tissue damage), various pathways are activated at the cellular level [5].

At the molecular level, trauma elicits both endogenous (DAMPs—Damage-Associated Molecular Patterns) and exogenous (PAMPs—Pathogen-Associated Molecular Patterns) inflammatory responses. DAMPs involve an exaggerated (hyperinflammatory) or insufficient (hypoinflammatory) host response to injury, whereas PAMPs originate from pathogens such as open traumatic wounds, infected tissues, or released toxins (e.g., sepsis caused by intestinal ischemia and microbial translocation into the bloodstream). Both DAMPs and PAMPs can activate similar inflammatory cascades, potentially leading to systemic inflammatory responses that complicate multiple trauma [5].

The individual immune response to hemorrhage plays a pivotal role in the pathophysiology of multiple trauma. In a study by McKinley et al., two groups of patients with comparable injury severity were analyzed in terms of tolerance to hemorrhagic shock. The researchers concluded that in “tolerant” patients—those in whom large-volume bleeding caused only minimal organ dysfunction—there was an early, significantly stronger immune response (involving a cluster of six cytokines: interleukin-9, 17E/25, 21, 22, 23, and 33, all highly interrelated) compared to patients sensitive to shock [8].

IL-6 levels have been shown to correlate with injury severity scores and to predict complications more reliably than many other inflammatory markers, largely due to its early elevation and sustained presence in circulation [9]. Furthermore, IL-6 measurement may inform surgical decision-making, particularly in selecting candidates for damage control strategies versus definitive care [9]. A major challenge in developing effective immunological damage-control therapies for traumatic hemorrhage (TH) is the lack of animal models that accurately reproduce the immune and pathophysiological responses observed in humans [10].

**Figure 1:**
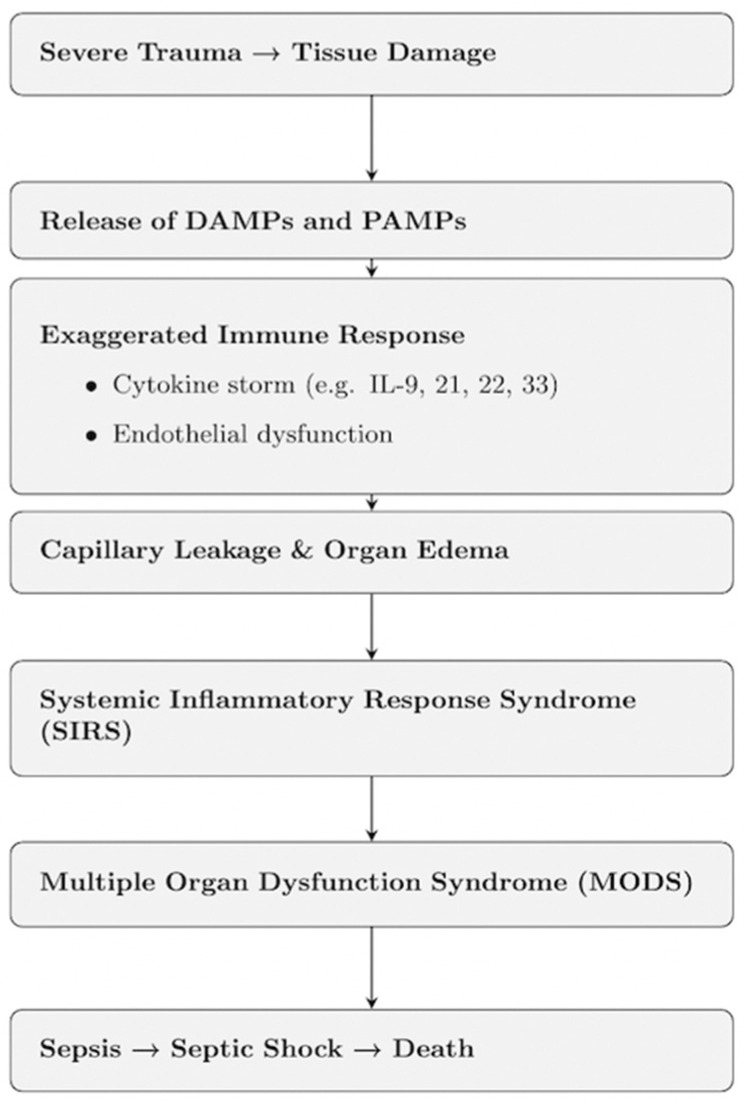
Pathophysiological Cascade in Multi-Organ Trauma. The scheme illustrates the immune and systemic response leading from trauma to MoDs and death.

In this context, the physiological response to trauma can be divided into early and late physiological responses to trauma. In the early phase, the Systemic Inflammatory Response Syndrome (SIRS) develops—a generalized response to stimuli such as trauma, burns, or infection. A key aspect of SIRS is uncontrolled endothelial damage and increased permeability, manifesting as organ dysfunction. During SIRS, organ edema (due to endothelial dysfunction) and cytokine storm (triggered by inflammatory mediators) occur. Importantly, SIRS does not require the presence of infection, but in the presence of bacteria, it may progress to sepsis. In the natural course of SIRS, it may further evolve into MODS—Multiple Organ Dysfunction Syndrome—a late systemic response. MODS is defined as dysfunction of at least two organs following trauma/shock/infection. Eventually, MODS may lead to sepsis, hemodynamic instability, and septic shock, which is a direct cause of death in patients with multiple trauma [5] (Figure 1).

### 2.3. Endothelial Dysfunction and Its Impact on Organ Function

In the context of multiple trauma, endothelial dysfunction warrants particular emphasis due to its central role in systemic pathophysiology. Injury to the endothelium increases capillary permeability, leading to fluid accumulation in the interstitial compartments of virtually all organ systems. In the lungs—an organ vital for efficient gas exchange—this process manifests as ARDS, wherein the presence of interstitial fluid disrupts alveolar-capillary oxygen transfer. In the gastrointestinal tract, ischemia coupled with heightened endothelial permeability promotes the translocation of endotoxins and microbial dysbiosis, thereby amplifying the systemic inflammatory response. Accumulation of fluid in the extravascular space further compromises tissue perfusion and impairs oxygen delivery, resulting in cellular hypoxia. These processes collectively contribute to multiorgan failure and are closely associated with delayed wound healing and skin integrity breakdown [5].

### 2.4. Diamond of Death—Interactions Between Components—The Impact of Blood Loss on Organ Function

The primary objective of DCR is the early and aggressive prevention and management of the pathophysiological components of the so-called “diamond of death,” a term used to describe the fatal cascade that often follows severe polytrauma [11,12,13,14].

Traditionally, this concept was framed as the “triad of death”—hypothermia, acidosis, and coagulopathy—each of which results primarily from massive haemorrhage and the associated resuscitative efforts.

Hypothermia frequently arises due to both blood loss and the administration of large volumes of unheated intravenous fluids. Lactic acidosis develops as a consequence of hypovolemia and tissue hypoxia, driving a metabolic shift toward anaerobic pathways and the subsequent accumulation of lactic acid. Coagulopathy is attributed to the dilutional and consumptive loss of clotting factors and platelets, in combination with impaired enzymatic function at lower core temperatures [11,12].

**Figure 2:**
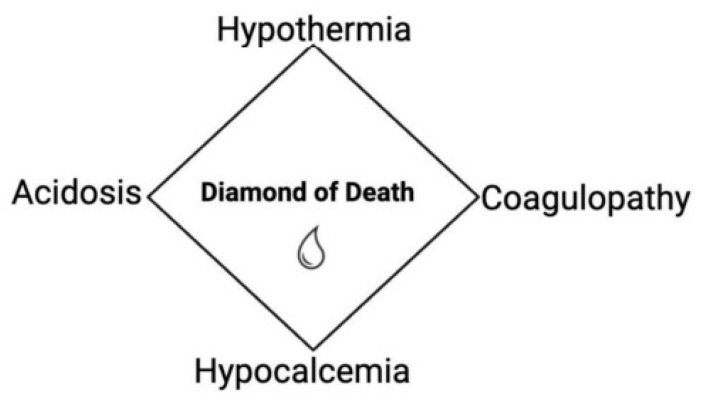
The “Diamond of Death” in trauma: interrelated pathophysiological components-hypothermia, acidosis, coagulopathy, and hypocalcemia—forming a self-reinforcing cycle in hemorrhagic shock.

More recently, hypocalcemia has been recognized as a fourth critical component, extending the triad into the more comprehensive “diamond of death” (Figure 2). Hypocalcemia in trauma patients is linked to increased mortality and arises both as a direct result of hemorrhage and as a consequence of its treatment. Mechanisms contributing to hypocalcemia include parathyroid gland dysfunction, decreased parathyroid hormone (PTH) secretion, transcellular calcium shifts, and the chelation of calcium ions by lactate and citrate—commonly present in transfused blood products [11,12].

The interplay between these components creates a self-reinforcing, deleterious cycle. For instance, correction of hypovolemia using blood products containing citrate may exacerbate hypocalcemia. Hypothermia impairs citrate metabolism, further worsening calcium depletion. Concurrently, hypovolemia leads to hypotension, and when combined with hypocalcemia-induced myocardial depression, results in reduced perfusion and worsening ischemia—thereby intensifying metabolic acidosis [7]. Due to the poor quality of studies concerning the citrate content in blood products used in transfusion, further research is necessary enabling the creation of practical recommendations regarding calcium supplementation in the care of the polytrauma patient [15]. The independent role of the calcium level in early mortality in polytrauma patients is challenged, citing a lack of statistically significant differences in mortality in patients requiring blood transfusion (at least 1 unit of red blood cells (RBC)) comparing the lethal diamond to the lethal triad [16]. Although there is no definitive Randomized Controlled Trial evidence, calcium supplementation is recommended in early care, attempting to counteract the mechanisms leading to the diamond of death [13,14].

### 2.5. Causes of Death in Multiple Trauma

The primary causes of early death in multiple trauma are massive hemorrhage and head injuries. Numerous randomized and observational studies indicate that hemorrhagic deaths typically occur between the 3rd and 6th h after injury. Between 6 and 24 h post-injury, traumatic brain injury is the most common cause of death, while multiple organ failure (MOF) becomes the leading cause of death in the first week after trauma [5].

## 3. Main Strategies of DCR (Figure 3)

One of the key pillars of the DCR strategy is the limitation of aggressive fluid resuscitation, which was commonly used in traditional trauma care models. However, the excessive crystalloids administration within the first 24 h post-injury has been associated with increased mortality and prolonged duration of mechanical ventilation. Additionally, variables such as advanced age, elevated ISS, and body temperature upon admission have been shown to correlate with in-hospital mortality, further underscoring the need for a judicious and individualized approach to fluid management in severely injured patients [17]. Although crystalloids and gelatin-based solutions may transiently support coagulation, their benefits diminish significantly once blood dilution exceeds 40%, at which point dilutional coagulopathy typically develops. When combined with hypothermia, this condition markedly increases the risk of uncontrolled haemorrhage [18]. Furthermore, excessive crystalloid administration has been associated with increased morbidity, as well as prolonged stays in both the intensive care unit and the hospital, particularly among patients with blunt trauma [19].

In response to these hemodynamic challenges, the DCR strategy incorporates permissive hypotension, also known as hypotensive resuscitation—an approach that deliberately maintains a lower mean arterial pressure (MAP) in the range of 50–60 mmHg. This technique has been shown to reduce mortality in patients experiencing hypovolemic shock [20]. By limiting hydrostatic pressure, it also minimizes ongoing hemorrhage, hemodilution, and subsequent tissue hypoxia [21]. Notably, one comparative study demonstrated that hypotensive resuscitation, when compared to traditional fluid strategies, results in improved 30-day survival and may offer a more cost-effective option, particularly in patients with blunt trauma [22].

### 3.1. Early Blood and Blood Product Transfusion

The early administration of blood products in a 1:1:1 ratio—comprising red blood cell concentrate (RBC), fresh frozen plasma (FFP), and platelet concentrate—has become a foundational principle of modern trauma resuscitation. In patients transfused with 6 to 10 units of RBC, no significant correlation was observed between the RBC-to-platelet ratio and 4 h mortality. However, an RBC-to-FFP ratio equal to or exceeding 4:1 was associated with a significantly increased risk of death compared to a balanced 1:1 ratio. In those receiving more than 10 units of RBC, each incremental increase in the RBC-to-FFP or RBC-to-platelet ratio was independently associated with higher mortality [23].

Although whole blood (WB) transfusion has not demonstrated a clear survival benefit over component therapy, it may offer logistical and operational advantages, particularly in prehospital and military environments [24]. Conversely, “balanced resuscitation”—defined as maintaining a component-based transfusion strategy with proportional ratios of RBC, FFP, and platelets—yields comparable mortality outcomes to WB and remains a practical standard in most civilian trauma systems [25].

### 3.2. The Role of Factor VIIa and Fibrinogen in Hemorrhage Control

Recombinant factor VIIa (rFVIIa) has demonstrated a significant reduction in the need for red blood cell (RBC) transfusions in patients with severe blunt trauma, while maintaining a favorable safety profile [26]. However, other studies do not consistently support its routine use, with findings suggesting potential adverse effects, as indicated by lower Glasgow Coma Scale (GCS) scores at discharge [27]. Similarly, the efficacy of fibrinogen concentrate (FC) and prothrombin complex concentrate (PCC) supplementation appears limited. Although both interventions reduce transfusion requirements and related complications, their impact on mortality remains inconclusive, highlighting the need for further research [28]. The use of these agents should, therefore, be considered on a case-by-case basis, taking into account patient-specific factors and the availability of diagnostic tools such as rotational thromboelastometry (ROTEM) or thromboelastography (TEG).

### 3.3. Early Hemorrhage Source Control

Damage control surgery (DCS) plays a pivotal role in the rapid control of massive hemorrhage, limiting ischemic damage, and improving survival rates. The introduction of temporary intravascular shunts (TIVS) has proven effective in reducing the time to limb revascularization and decreasing the risk of amputation [29,30]. In DCS procedures, laparotomy and the use of temporary vascular clamps or shunts further minimize limb ischemia and reduce the likelihood of amputations [31]. Such temporary interventions, particularly TIVS, serve to significantly shorten revascularization times, thereby enhancing the chances of limb salvage [32].

### 3.4. The Importance of Early Embolization

Intravascular interventions, such as early embolization, have gained significant traction in the management of traumatic hemorrhages. In cases of spleen injuries, prophylactic embolization has been shown to reduce mortality, decrease the need for surgical interventions, and minimize complications [33]. Conversely, delayed embolization in pelvic fractures has been associated with significantly higher mortality rates [34].

### 3.5. Transport Time and Survival

Prehospital time plays a critical role in determining trauma treatment outcomes. Shorter transport times, particularly in patients with penetrating injuries and central nervous system (CNS) trauma, have been shown to significantly reduce mortality rates [21]. Although the effect of prehospital time on outcomes is well established, the impact may vary depending on the healthcare system in place. Notably, a study conducted in Korea found that longer scene times were associated with lower mortality, highlighting the influence of healthcare infrastructure and trauma care protocols in different regions [35].

### 3.6. Preventing the Death Triad: Hypothermia, Acidosis, Coagulopathy

DCR prioritizes the active prevention of hypothermia, a critical factor that can exacerbate coagulopathy. Immediate removal of wet clothing, combined with the application of both active and passive warming techniques, effectively restores core body temperature and enhances patient comfort [36]. Importantly, active warming methods have been demonstrated to carry no significant adverse effects [37].

Regarding acidosis, routine administration of bicarbonates is generally discouraged and should be reserved for cases of severe metabolic acidosis (pH < 7.2) [37]. Excessive dosing or rapid infusion carries risks of metabolic derangements, including hypernatremia, decreased ionized calcium, and accelerated lactate production. Nevertheless, in select patient populations—such as those with hyperchloremia—bicarbonate therapy may confer prognostic benefit [37,38].

### 3.7. TXA

TXA, according to the CRASH-2 trial, reduces mortality from 5.7% to 4.9% due to bleeding. When used within an hour of injury, it reduces the risk of death from 7.7% to 5.3%. If administered within 3 h of injury, mortality is reduced from 6.1% to 4.8% Treatment after 3 h appeared to increase the risk of death from bleeding from 3.1% to 4.4% [39]. Notably, TXA does not raise the risk of thrombotic complications, and it has been shown to decrease the need for blood product transfusions, ultimately reducing treatment costs [40]. While its impact on long-term functional outcomes has not been definitively proven, its use in the early phase of trauma seems justified [41].

**Figure 3:**
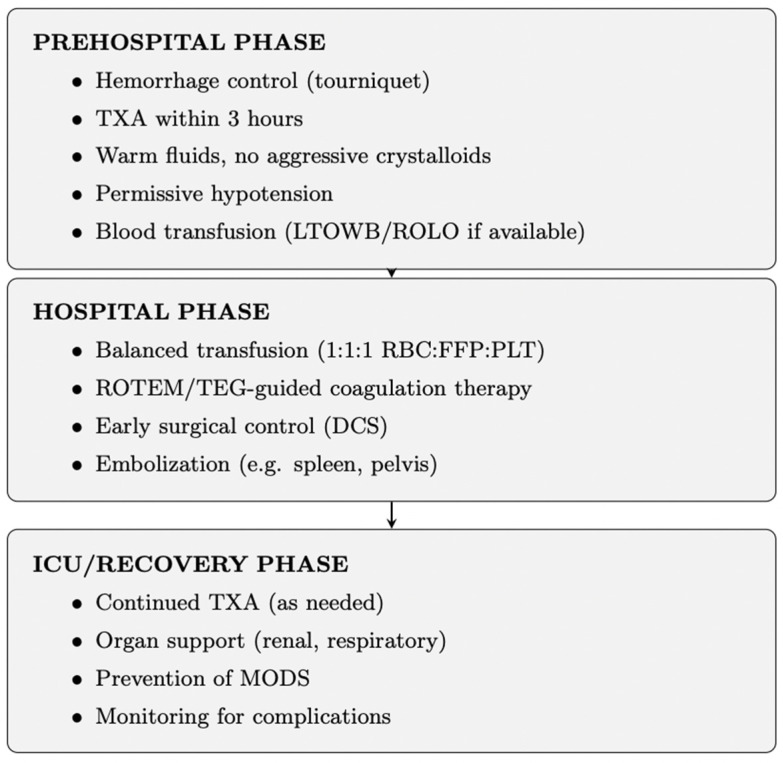
Phased DCR Strategy in Trauma Care. The diagram illustrates interventions from prehospital through ICU phases.

## 4. Application of “Damage Control” in Prehospital Conditions

Prehospital care is a crucial component in providing assistance to patients with multiple trauma injuries. It primarily involves hemorrhage control, blood transfusions, and pain management [42].

### 4.1. Hemorrhage Control

**Figure 4:**
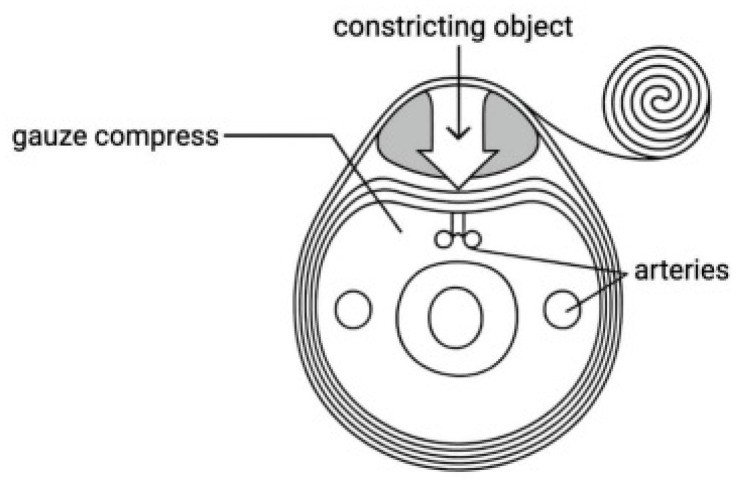
Application of a compression bandage as part of hemorrhage control in the prehospital phase of Damage Control Resuscitation (DCR).

In a landmark 10-year analysis of battlefield data, Eastridge et al. reported that 24.3% of all combat deaths were potentially avoidable, with hemorrhage control identified as the critical factor capable of preventing 90.9% of these fatalities [43]. After prolonged debate regarding tourniquet use in battlefield medicine [44,45], current evidence supports their invaluable role in prehospital hemorrhage management during life-threatening scenarios [46]. Meléndez-Lugo et al. further endorse tourniquets as a primary intervention for bleeding control, recommending that fluid resuscitation begin with minimal crystalloid administration, adhering to permissive hypotension principles. To mitigate hypothermia, the application of warm blankets alongside warmed intravenous fluids is essential. Importantly, prehospital procedures must not delay definitive transport to medical facilities [47] (Figure 4).

**Figure 5:**
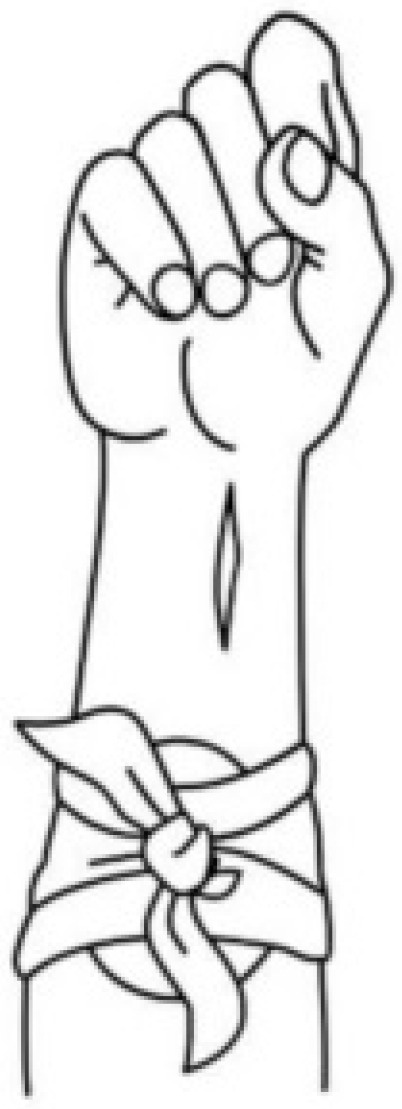
Compression bandage application technique to the forearm in prehospital trauma care.

A multicenter analysis evaluating the use of tourniquets in severe limb trauma compared patients who received tourniquets with a matched control group without tourniquet application. Both cohorts were comparable in terms of injury severity and vital signs upon presentation. The study demonstrated that tourniquets effectively achieved hemostasis in 87.7% of affected limbs. Despite sustaining more severe limb injuries, patients treated with tourniquets were less likely to present to the hospital in shock compared to controls. Crucially, the incidence of complications, such as limb ischemia, did not differ significantly between the groups. These findings affirm the safety of tourniquets and support their widespread implementation to improve outcomes in patients with severe extremity injuries [48] (Figure 5).

It is important to acknowledge, however, that the use of tourniquets is not without risks. Holcomb et al. highlighted that, when applied correctly (“high and tight”), tourniquets left in place for more than two hours—especially when evacuation or advanced medical care is delayed—pose significant risks, including limb ischemia, muscle necrosis, AKI, amputation, and even mortality. Therefore, timely reassessment and potential replacement of the tourniquet are critical to mitigate these complications. Historical overuse of tourniquets, notably during World War II, led to prolonged application without adequate monitoring, contributing to avoidable adverse outcomes. Consequently, regular evaluation of tourniquet necessity is essential. Previously applied TQ should be convert or replace [49].

### 4.2. Blood Transfusion

An essential element of prehospital resuscitation is blood transfusion, with low-titer group O whole blood (LTOWB) representing the optimal choice due to its balanced hemostatic profile and minimized risk of alloimmunization. In circumstances where LTOWB is not available, transfusion of fresh whole blood with a low-titer group O (ROLO), commonly sourced from ambulatory donors in combat or austere environments, constitutes a practical and effective alternative [42].

### 4.3. Pain Management

Historically, morphine and fentanyl have been the mainstay analgesics in prehospital pain management. However, contemporary practice increasingly favors agents such as ketamine and inhaled methoxyflurane due to their rapid onset and ease of administration. Despite these advantages, both agents present limitations; ketamine, in particular, remains contentious because of its dissociative properties and the potential to induce hallucinations, raising concerns regarding its safety profile in the prehospital setting [42,43,44,45,46,47,48,49,50,51].

### 4.4. Impact of Transport Modality and Trauma Center Level on Survival Outcomes in Severely Injured Patients: The Critical Role of HEMS in DCR

In a comprehensive study conducted in Sweden by Lapidus et al., survival outcomes of trauma patients transported via HEMS were compared to those transported by Ground Emergency Medical Services (GEMS). The cohort was stratified according to ISS into three categories reflecting injury severity: ISS 9–15, ISS 16–24, and ISS ≥ 25. The findings revealed a statistically significant reduction in mortality among patients transported by HEMS across all ISS categories, with the mortality differentials widening concomitantly with injury severity (1.9% vs. 4.3% for ISS 9–15, 5.4% vs. 9.4% for ISS 16–24, and 31% vs. 42% for ISS ≥ 25) (Table 1).

Nevertheless, HEMS transport was paradoxically associated with poorer neurological outcomes, prolonged time to antibiotic administration (TTA), and increased frequency of prehospital intubation. Notably, although prehospital times were longer for HEMS compared to GEMS, survival benefits were consistently observed in all ISS strata. These data underscore the imperative to expand HEMS availability, reinforcing its preferential role in the triage and transport of severely injured patients, particularly within the framework of implementing DCR strategies [52].

In a study conducted by Zadorozny et al., the influence of transport modality— HEMS versus GEMS—on the relationship between prehospital time and in-hospital treatment outcomes was examined. The authors reported that patients transported by HEMS experienced longer prehospital times, which correlated with an increased likelihood of receiving prehospital blood transfusions compared to those transported by GEMS. This disparity is likely attributable to the greater severity and instability of injuries among patients transported via HEMS [53].

Moreover, the study found that, independent of transport type, prolonged prehospital time was paradoxically associated with reduced mortality, suggesting that patients enduring longer transport intervals were more stable and benefitted from effective prehospital management. The nature of the injury further modulated these outcomes. Specifically, in cases of severe isolated thoracic trauma transported by HEMS, extended prehospital times were linked to an increased probability of receiving transfusions upon hospital admission. Conversely, for patients with polytrauma, longer prehospital times during GEMS transport were associated with decreased mortality. These findings imply that a prolonged, yet optimally managed, prehospital phase may confer a survival advantage over more expedited, but potentially less controlled, transport strategies [53].

In a study conducted by Dinh et al. in Wales involving 9012 patients, transport times from suburban versus urban areas were compared. Transport from suburban areas (*n* = 3071) averaged 95 min, significantly longer than transport from urban areas (*n* = 5941), which averaged 65 min. Despite this notable difference in transport duration, no statistically significant difference in 30-day mortality was observed between the two groups [54].

Similarly, Nabeta et al. concluded that HEMS provide comparable treatment opportunities and effectively minimize trauma-related mortality for patients transported over greater distances to trauma centers when compared to GEMS [55].

The objective of the study by Sewalt et al. was to ascertain which patient cohorts benefit most substantially from direct transfer to Level 1 trauma centers, recognized as the apex of trauma care facilities. Their analysis revealed a modest but statistically significant survival advantage associated with direct admission to Level 1 centers. Conversely, comparative assessment between Level 1 or Level 2 trauma centers and Level 3 centers demonstrated no appreciable differences in patient outcomes. Importantly, the data suggest that individuals presenting with traumatic brain injuries and hemodynamic instability are more likely to derive meaningful benefit from the specialized resources available at Level 1 trauma centers [56].

According to a large-scale study by Deeb et al., encompassing over 36,000 trauma patients, direct transport via HEMS to a trauma center was associated with nearly a twofold increase in survival compared to initial admission at smaller, non-trauma facilities. The most pronounced survival advantage was observed among patients presenting in critical condition—characterized by a Glasgow Coma Scale (GCS) score below 13, hypotension, abnormal respiratory rates, paralysis, severe thoracic trauma, and multi-organ injuries [57].

Synthesizing findings from these studies, it becomes evident that the decision to proceed with direct transport to a specialized trauma center versus initial stabilization at a local hospital hinges on several pivotal factors. These include the patient’s clinical status, the availability and type of transport resources, and the distance to the nearest trauma center. Current evidence underscores the imperative to minimize prehospital time and advocates for HEMS as the preferred transport modality, particularly over extended distances. Importantly, the aggregate data suggest that transport duration alone is not the sole determinant of patient outcomes; rather, the quality of prehospital medical care—typically superior with HEMS—is paramount. Furthermore, there is a pressing need to refine triage protocols to ensure that the most severely injured patients are promptly routed to facilities equipped with the highest level of care [52,53,54,55,56,57].

## 5. Review of Scientific Studies and Current Guidelines

In recent years, a growing body of clinical evidence has confirmed the effectiveness of DCR strategies in improving survival among trauma patients. The application of tactical tourniquets has been identified as an independent predictor of increased survival [58]. Moreover, the implementation of viscoelastic assays—such as thromboelastography (TEG) and thromboelastometry (ROTEM)—in the assessment of coagulation status has been associated with improved patient outcomes [59]. The use of frozen whole blood, as well as the initiation of prehospital blood transfusion, has similarly demonstrated a positive impact on survival rates, particularly by increasing the proportion of patients who survive to hospital admission [60,61].

According to current Advanced Trauma Life Support (ATLS) guidelines, there has been a paradigm shift away from aggressive crystalloid resuscitation toward DCR principles, which prioritize the early administration of blood products in balanced ratios. This approach aims to disrupt the “lethal triad” of hypothermia, acidosis, and trauma-induced coagulopathy, thereby improving outcomes in severely injured individuals [62].

The European Resuscitation Council (ERC) guidelines further reinforce this strategy, recommending a limitation of crystalloid use to no more than 20 mL/kg and advocating for the prompt initiation of blood component therapy. These guidelines also underscore the timely administration of TXA, particularly in pediatric patients, who should receive it within the first three hours post-injury. Additionally, the application of permissive hypotension is considered acceptable in children, provided there is no clinical suspicion of traumatic brain injury [63].

## 6. Summary and Conclusions

Research results show that advanced hemorrhage control techniques significantly increase patient survival, particularly in pre-hospital settings. Using synthetic oxygen carriers can also significantly impact patient survival and recovery when red blood cell concentrate is unavailable. Methods that minimize damage to erythrocytes and enable blood storage in prehospital settings also appear promising. Modern hemostatic agents are highly effective and often play a crucial role in saving patients’ lives and health in cases of severe bleeding. Unfortunately, the current cost of these preparations prevents them from being used as basic equipment in every ambulance or among soldiers.

## Figures and Tables

**Table 1:** Mortality Rates in Trauma Patients Transported by HEMS Versus GEMS, Stratified by ISS.

ISS	Mortality HEMS	Mortality GEMS	Difference (%)
9–15	1.90%	4.30%	2.4
16–24	5.40%	9.40%	4
≥25	31%	42%	11

## Data Availability

No new data were created or analyzed in this study. Data sharing is not applicable to this article.

## Abbreviations

The following abbreviations are used in this manuscript:

REBOA	Resuscitative Endovascular Balloon Occlusion of the Aorta
AOCs/HBOCs	hemoglobin-based oxygen carriers
SMP	shape-memory polymer
SCA	Sudden cardiac arrest
CPR	Cardiopulmonary
ISS	Injury Severity Score
LAA	London Air Ambulance
PFD	Perfluorodecalin
RBC	Red blood cell
AOCs	Artificial Oxygen Carriers
DCR	Damage Control Resuscitation
ARDS	Acute Respiratory Distress Syndrome
AKI	Acute Kidney Injury
ACS	American College of Surgeons
ROTEM	Rotational thromboelastometry
TEG	Thromboelastography
TXA	Tranexamic Acid
HEMS	Helicopter Emergency Medical Services
AIS	Abbreviated Injury Scale
DAMPs	Damage-Associated Molecular Patterns
PAMPs	Pathogen-Associated Molecular Patterns
SIRS	Systemic Inflammatory Response Syndrome
MODS	Multiple Organ Dysfunction Syndrome
PTH	Parathyroid Hormone
MOF	Multiple Organ Failure
MAP	Mean Arterial Pressure
FFP	Fresh Frozen Plasma
WB	Whole Blood
rFVIIa	Recombinant Factor VIIa
GCS	Glasgow Coma Scale
FC	Fibrinogen Concentrate
PCC	Prothrombin Complex Concentrate
DCS	Damage Control Surgery
TIVS	Temporary Intravascular Shunts
CNS	Central Nervous System
LTOWB	Low-titer Group O Whole Blood
